# Flexible foraging strategies in *Pipistrellus pygmaeus* in response to abundant but ephemeral prey

**DOI:** 10.1371/journal.pone.0204511

**Published:** 2018-10-04

**Authors:** Lucinda Kirkpatrick, Jennifer Graham, Sean McGregor, Lynn Munro, Matheus Scoarize, Kirsty Park

**Affiliations:** 1 Biological and Ecological Sciences, University of Stirling, Scotland; 2 EVECO, Universiteit Antwerpen, Antwerp, Belgium; 3 WDC, Scottish Dolphin Centre, Spey Bay, Fochabers, Moray, Scotland; 4 Biophore, University of Lausanne, Lausanne, France; 5 PEA, Universidade Estadual de Maringá, Maringá, Paraná, Brazil; Sichuan University, CHINA

## Abstract

There is growing recognition that with sympathetic management, plantation forests may contain more biodiversity than previously thought. However, the extent to which they may support bat populations is contentious. Many studies have demonstrated active avoidance of coniferous plantations and attributed this to the lack of available roost sites and low invertebrate density. In contrast, other work, carried out in plantation dominated landscapes have shown that certain bat species are able to exploit these areas. However, the extent to which bats use plantations for roosting and foraging, or simply move through the plantation matrix to access more favourable sites is unclear. We radio tracked female *Pipistrellus pygmaeus* over two summers to establish the extent to which individual bats use Sitka Spruce plantations in southern Scotland for foraging and roosting and assess the implications for felling operations on bats. Maternity roosts identified (n = 17) were in all in buildings and most were large (> 500 individuals). We found no evidence of bats roosting in mature Sitka Spruce crop trees, although several bats used roosts in old or dead beech and oak trees as an alternative to their main maternity roost. Home ranges were much larger (mean 9.6 ± 3.12 km^2^) than those reported from other studies (0.6–1.6 km^2^), and it is likely that roost availability rather than food abundance constrains *P*. *pygmaeus* use of Sitka Spruce plantations. At the landscape scale, most individuals selected coniferous habitats over other habitat types, covering large distances to access plantation areas, whilst at a local scale bats used forest tracks to access water, felled stands or patches of broadleaf cover within the plantation. Sitka Spruce plantations support a high abundance of *Culicoides impuctatus*, the Highland midge which may act as a reliable and plentiful food source for females during lactation, an energetically expensive period. The use of felled stands for foraging by bats has implications for forest management as wind turbines, following small-scale felling operations, are increasingly being installed in plantations; wind turbines have been associated with high bat mortality in some countries. Decisions about siting wind turbines in upland plantations should consider the likelihood of increased bat activity post felling.

## Introduction

In landscapes where we lack a thorough understanding of the interactions and relationships between organisms and their environment, it can be difficult to manage anthropogenic environmental change for the benefit of biodiversity. For example, commercially managed plantation forests, planted with non-native tree species, are a widespread land use type in much of Europe. However, information on the impact that management has on both abiotic and biotic environments, and consequently the organisms which are present, is sparse for many taxa. Plantations are usually large in size, intensively managed and often under surveyed, perhaps due in part to the perception of such habitats as poor for biodiversity. As a result, sufficient information to determine the impact of management on organisms [1,2] or to assess the influence of changing management practices is often lacking. There is growing evidence that changing forest management practices can facilitate social and ecological benefits without impacting economic performance [2–5], thereby providing an opportunity to manage plantations in ways that benefit both biodiversity and commercial interests.

Commercial plantations in Europe are generally considered poor habitat for bats [1,6], which may have resulted in their potential contribution in supporting bat populations being under explored [5]. However, a growing body of research suggests that plantations can, if suitably managed, fulfil roosting and foraging requirements [7,8], at least for some bat species. For example, extensive bat use of commercial plantation forests has been reported from Australia [7,9,10], New Zealand [11,12], North America [13], Italy [14], France [15] and the UK [16,17]. Although these plantations differ greatly in terms of tree species and forestry practices, bats appear to respond broadly similarly to certain management prescriptions, for example increased bat activity in response to reduced stand density [9,13,14,17–19]. Despite this, forest plans currently lack the appropriate information to ensure management for bats is effective and meets legislative requirements [5]. Furthermore, plantations are increasingly being used for the installation of wind turbines. Turbines have been associated with bat mortality [20] but the extent to which keyholing in plantation areas may pose a risk has not been evaluated. The species most vulnerable to death or injury from wind turbines in the UK are *P*. *pygmaeus*, *P*. *pipistrellus* and *N*. *leisleri* [21], which are both abundant in our study area [17] and increase activity after felling [22]. However, bat fatalities in response to wind turbines can be highly variable and site specific; Evidence from North America that bat fatalities are likely to be higher in areas with reduced roosting resources, foraging opportunities and presence of migratory routes [23]. Therefore, understanding how bats use different areas of the plantation is essential for informing forest management plans and ensuring that appropriate mitigation is carried out if wind turbine installation is intended.

Forests are a critical habitat for most bat species, both for foraging and roosting [24]. In addition, forests can provide protection from predators, and provide linear features to allow easier negotiation around the landscape [25–27]. The extent to which commercial plantations can fulfil bats’ requirements varies; conifer plantations are primarily planted with non-native, fast growing tree species which are harvested before reaching maturity, rarely developing features appropriate for bat roosts [28]. However, suitability as roosts varies between both tree and bat species. For example both *Pinus nigra* and *P*. *sylvaticus* were used as maternity roosts by colonies of *M*. *nattereri* in Scotland [16], but there is no evidence that *Picea sitchensis* has been used by bats for either day roosts (temporary roosts used by a small number of bats) or maternity roosts (roosts used by females and their young, which are often used annually and can contain large numbers of bats). Felling may directly cause mortality through the removal of roost trees if occupied by bats, or indirectly by reducing the reproductive potential of a population [11,28,29]. In addition, the practice of clear felling, where large scale removal of trees of harvestable age can result in extreme habitat alteration. The creation of such large gaps in forest cover can potentially limit bat movement around plantation landscapes [30] or, alternatively, open up new foraging areas [22,31]. Finally, while plantations may support substantial invertebrate populations, the increased structural complexity in densely planted plantation forests may limit access to invertebrate prey for all but the most manoeuvrable bat species [17,32,33].

Many bat species in Western Europe have undergone severe population declines in the previous decades [34] although monitoring programs have shown that some populations are beginning to recover as a result of increased protection [35]. In the United Kingdom, seven of the 16 resident bat species, including *Pipistrellus pygmaeus*, are listed on the UK Biodiversity Action Plan.

Recent studies have found that bat activity in Sitka Spruce plantations was dominated by *Pipistrellus spp*., particularly *P*. *pygmaeus*. This was despite previous studies suggesting that *P*. *pygmaeus* often avoids commercial plantations and favours riparian habitats [36–38], possibly due to low invertebrate densities [37]. However, *P*. *pygmaeus* preferentially feeds on nematoceran Diptera with aquatic larvae [39], which are abundant in Sitka Spruce dominated plantation landscapes, particularly the Highland midge (*Culicoides impuctatus*). This suggests that commercial plantations may be more appropriate for *Pipistrellus spp*. than previously considered. Furthermore, we previously found that the majority of *P*. *pygmaeus* trapped in Sitka Spruce plantations were lactating females, indicating use of plantation forests during an energetically expensive period [17]. *Pipistrellus pygmaeus* preferentially forms maternity colonies in buildings and is less dependent on tree cavity roosts than other bat species, which are often lacking in plantation landscapes. However, the extent to which *P*. *pygmaeus* associate with particular habitats within plantations, and whether they roost in tree cavities in mature conifers (which may put them at risk from felling operations) is currently unknown. Specifically, in this study, we aimed to:

1. Identify maternity and day roosts for *P*. *pygmaeus* in plantation landscapes
2. Characterise bat habitat associations within plantation landscapes at multiple spatial scales
3. Identify key foraging habitats
4. Use the findings to make management recommendations

## Methods

### Study area, colonies and capture method

The study was conducted between early June and late August in 2014 and 2015 within Galloway Forest Park in South west Scotland. Galloway Forest Park is a large (114,000 ha), upland, coniferous plantation dominated by *Picea sitchensis* (Sitka Spruce), managed primarily for timber extraction. Following widespread deforestation during the Holocene, Galloway consisted of open uplands with a few, isolated patches of broadleaf woodland. After 1925, intensive planting of commercial conifers created the current landscape of stands (a forestry unit denoting an area of even aged trees, usually planted at the same time) of conifer at various ages and densities interspersed with open uplands and small patches of fragmented broadleaf woodlands. Several small lochs and rivers are scattered throughout the landscape. Bats were trapped at foraging sites within the plantation (see Table A in S1 Appendix for a description of field sites) where acoustic surveys were being conducted as part of another study [40]. All trapping sites were within the plantation boundary, and within 4km of a patch of broadleaf woodland.

All trapping sessions began 30 minutes after midnight to reduce the likelihood of catching commuting bats, as we wanted to target bats foraging within plantation areas. Individual bats were trapped by placing three six metre mist nets (Ecotone, PL) and a harp trap (Austbat, Aus) across potential flight-lines in plantations. We used an acoustic lure (Sussex Autobat; [41]) with four different synthesised calls played for 15 minutes at each net which has been shown to increase capture rates [42]. After capture, bats were held in bags before biometric data was recorded. We recorded mass to an accuracy of 0.1g and forearm to 0.1mm. Individuals were aged based on ossification of the phalangeal joints and sexed [43]. We assessed the reproductive status of the females we trapped by the presence of hairless, large nipples and whether they were palpably parous. We stopped trapping during late June when females are likely to be heavily pregnant to reduce the stress of catching. Only females were used for the tracking study as we were primarily interested in how *P*. *pygmaeus* uses plantations during pregnancy and lactation, an energetically costly period. We selected individuals for trapping based on a minimum weight of 6.0 g [44] and reproductive status. One female juvenile was tagged in the first year as adult females began to disperse out of the plantation earlier than anticipated. No juveniles were captured in the second year. All bat handling, trapping and tagging was carried out under license (Scottish Natural Heritage; license numbers 19584 and 46169). Ethical approval was given by University of Stirling Biological and Environmental Sciences Ethical Committee.

### Transmitters and tracking methods

Bats were tagged with Holohil LB-2X (Holohil Systems, Carp, Ontario, Canada) VHF radio transmitters which are specifically designed for tracking small bat species. Tags weigh between 0.22–0.31g in weight and are the smallest tags currently available. The fur on the back of the bats between the scapulae was trimmed and transmitters were attached using a surgical latex cement (Torbot Ostomy and Medical Supplies, Rhode Island, US) which provides a flexible hold and limits disturbance to the bat. Transmitter batteries had a minimum life span of 7 days although several lasted 14 days. One tagged failed shortly after application and all others detached before battery failure, probably due to grooming. Bats were tracked using a combination of Sika (Biotrack, UK) and Australis (Titley, Australia) receivers with hand held Yagi aerials. The topography of the area, the density of plantation stands and the limited range of the tags meant that bats were located by “homing in”, a technique by which fieldworkers follow a signal’s increasing strength until the animal is observed, or circling a small area under the assumption that the animal is within the area[45]. Field workers worked in pairs, homing in on bat locations while another fieldworker used higher altitude positions to locate bats when they were lost. If contact with the bat was lost for more than half an hour then the night was not considered a full session; only full sessions were used for further analysis. Locations were assigned an accuracy based on confidence in the location of the bat (the quality of the signal and whether field workers observed the bat). The accuracy bands were determined by carrying out field experiments with a transmitter in different habitats. Tags were positioned at known points, and the strength of the signal at distances from this point were recorded. Fieldworkers were trained with these tags to ensure familiarity with the process of tracking before bats were tagged. Points with an estimated accuracy of < 100m were retained for further analysis. Bearings were recorded as frequently as necessary to ensure continuous contact with the bat, dependent on whether the bat was foraging (moving consistently within a small area, many bearings) or commuting (moving quickly from one area to another). Bearings were subsequently subsampled to every 5 minutes to reduce temporal autocorrelation.

Field workers recorded time, location of observers (ten figure grid reference), GPS waypoint, bearing on the bat, accuracy band, description of the location and notes about the bats behaviour and location (e.g. flight height, whether other bats were foraging nearby, habitat over which bats were foraging). Weather conditions such as temperature, rain and cloud cover were recorded at regular intervals. The positions of the bats were calculated from their bearing and estimated distance and re-projected as estimated locations using R (R core development team, version 3.3.1; [46]). Maternity roosts were determined by the building which the female returned to most regularly. Both day and night roosts were also recorded, often consisting of a tree or a derelict building within the plantation. Partial counts were carried out at maternity colonies and day roosts where possible (objective 1), but do not represent full counts as surveyors typically left before all bats finished emerging, to assist in locating tagged bats.

### Analysis of habitat

All analysis was carried out in R using the following packages: AdehabitatHS, AdehabitatHR, rgeos, raster, sp, rgdal, ggplot2. Habitat was assessed on two scales (objective 2). We used a broad resolution habitat map to distinguish between plantation and non-plantation areas. Next, we created a finer resolution map which classified habitats on a finer scale to identify the key plantation management stages bats associated most closely with. We removed open upland areas, as despite being within the plantation boundary, no foraging bearings were recorded within this area. We chose to use to separate maps to reduce the number of land classes we investigated for each resolution. Using the broad scale resolution map, we were able to identify whether individuals primarily used plantation areas or were simply moving through the plantation to access non-plantation habitats (objective 2). Using the fine scale resolution map, we were able to further refine our understanding of bat responses to different management prescriptions within the plantation boundary (objective 3) and relate this to specific management recommendations (objective 4). First, using the Centre for Ecology and Hydrology 2007 Land Cover Map [47] and a Forestry Commission specific database we created a *broad resolution* map by categorising the landscape according to the following variables: Buildings, Broadleaf, Conifer, Open (upland or moorland), Tracks, Mixed woodland (both broadleaf and conifer), Water, Grassland (including improved pasture). Second, using a Forestry Commission specific database, we refined our classification of plantation habitats. Stands were classified into felled (felled within 3 years of the sampling), young conifer (stands less than 10 years old) and closed canopy conifer (stands more than 25 years old; Table A in S1 Appendix). We included patches of broadleaf at a finer scale than that of the land cover map which often classified mixed or broadleaf patches as conifer if they were within the plantation boundary, capturing remnant patches remaining within the conifer plantation. We also included rivers and bodies of water.

Ranges of tagged bats were calculated using two methods. Home range was determined by using a 95% minimum convex polygon (MCP) around all locations, and core areas, where bats spent the most time (objective 3), were determined using an 80% kernel density estimate (KDE: density estimate of 80% of activity) with a smoothing factor of 83 (the standard deviation of the estimated accuracy) and the same grid for all animals [48]. Least squares cross validation [49] was unsuitable in this case as different animals required different smoothing parameters, which render comparisons between individuals meaningless. To evaluate the effects of year, reproductive status of the individual, and the location of its roost (within or outside the plantation) on home range sizes (objective 4), we used two-way analysis of variance (ANOVA). Two-way ANOVAs were also used to compare the number and duration of foraging bouts between years and reproductive status of bats. Finally, the effect of temperature (minimum temperature during the tracking period) on several foraging metrics (number and duration of foraging bouts, and the furthest Euclidean distance travelled each foraging session) was investigated using multiple linear regressions. (objective 4). Residuals were checked to ensure assumptions of normality were met. Since there was no statistically significant difference in core area or home range between the two sample years (CA: F_1,9_ = 0.76, p = 0.41; HR: F_1,9_ = 0.73, p = 0.41) or between bats of differing reproductive status (CA: F_2,8_ = 0.46, p = 0.64; HR: F_2,8_ = 1.724, p = 0.23), we pooled telemetry data across year and reproductive status to calculate means of home range area and core area [50].

Habitat associations were assessed on two scales. At the landscape scale the proportion of each habitat in the individual bats MCP was compared to the available habitat, which was determined by calculating an MCP around all tracking locations for all bats (second order habitat selection; [51]). On the local scale we compared the selection of habitats within each individual bat’s home range to the selection of habitat within their core area as determined by kernel density estimation (third order habitat selection; [51]). We used χ tests to assess whether habitat selection was consistent across individuals, and as it was not, used selection ratios [52] to assess individual associations with habitat types. Graphical exploration of eigen analysis of selection ratios [53] provided further clarification on the direction and magnitude of habitat selection. This approach was applied to habitat selection using both the *broad habitat* map and the finer resolution forest management map at both spatial scales. Manly selection ratios were used to investigate individual bat selectivity. A Manly selection ratio of close to 1 is indicative of no selection, below 1 indicates avoidance of the habitat and above 1 indicates selection of the habitat in relation to its availability [52]. We ranked habitats by the number of bats positively selecting that habitats, “selective” bats were those for which 70% of the foraging fixes were in a single habitat type while “non-selective” individuals used a wider variety of habitats.

## Results

Eleven individual female *P*. *pygmaeus* (five in 2014, six in 2015) were radio tracked successfully for between 3 and 6 consecutive calendar days between June and August (Table 1). We collected a total of 9050 telemetry locations, which was reduced to 2371 after subsampling every five-minute intervals.

**Table 1:** Reproductive status, biometric details, tracking information and home range / core area details for individual *Pipistrellus pygmaeus*.

Animal ID	Study year	Reproductive status	Forearm (mm)	Mass (g)	Number of nights	Number of locations	Home range area (km^2^ MCP)	Core foraging area (km^2^)
3	2014	Lac	32.4	6.4	5	585	0.58	1.72
4	2014	Lac	32.5	6.0	3	627	32.38	13.36
6	2014	Lac	33.0	6.1	5	673	19.56	12.40
8	2014	Plac	33.0	6.4	5	974	9.58	7.55
9	2014	NA (Juvenile)	32.8	6.6	3	405	0.28	1.59
10	2015	Preg	32.0	6.7	4	910	7.36	5.41
11	2015	Preg	30.8	7.0	4	751	3.63	5.98
12	2015	Preg	31.3	7.2	4	649	4.24	6.12
14	2015	Lac	31.9	6.3	4	740	21.06	6.83
15	2015	Lac	32.5	6.4	6	1887	3.62	2.70
16	2015	PLac	31.9	6.5	5	849	2.29	4.57

**Fig 1:**
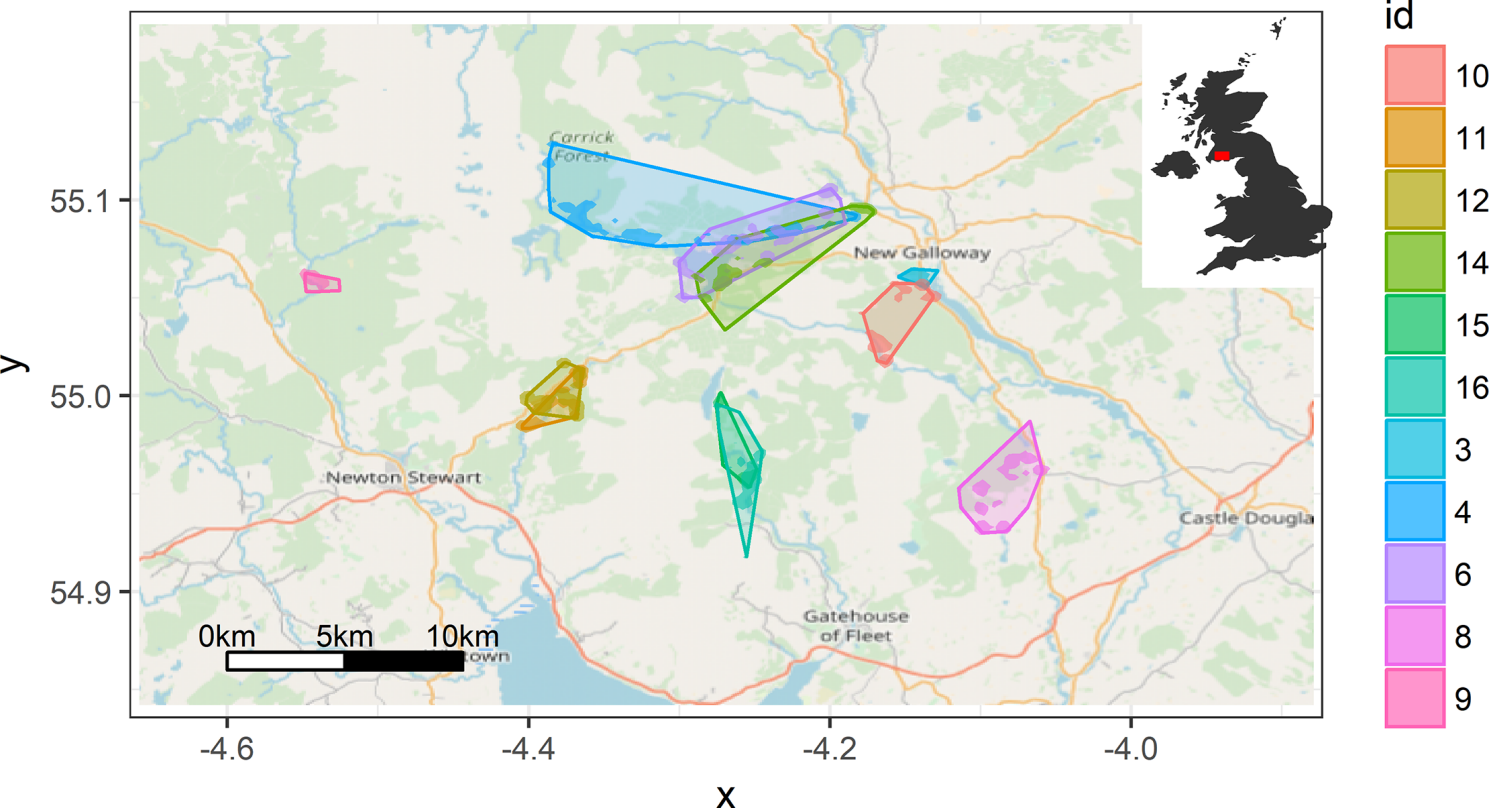
Map showing the home ranges of individual bats radiotracked in Galloway Forest Park as defined by (i) minimum convex polygons (light shaded area) and (ii) kernel density estimates (dark shaded area).

We identified 17 new maternity roosts from the tagged bats (Fig 1). Bats foraging in similar areas were often roosting in separate roosts, and switching between maternity roosts was low. The majority of roosts were in buildings, including all maternity roosts, although there was some diurnal use of tree roosts and one individual regularly used a derelict hut in the plantation interior as a day roost. All tree roosts were in old or dead deciduous trees and we found no evidence of roosting in Sitka Spruce despite this being the dominant tree species in the area.

### Spatial behaviour

**Fig 2:**
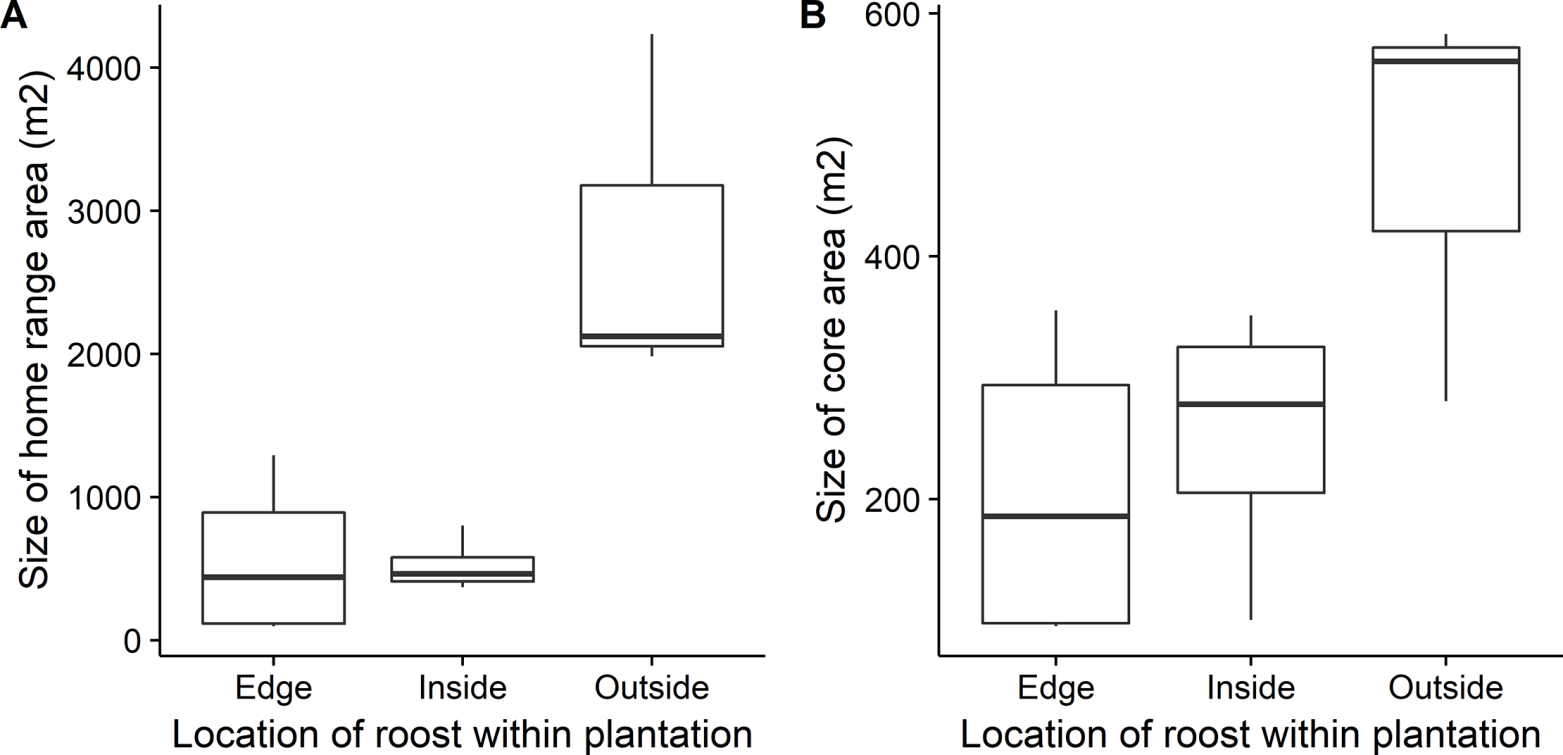
Difference in the size of the home range and core area as a function of roost location compared to the plantation boundary.

Mean home range (HR) area was 9.51 ± 3.12 km^2^ and was highly variable across individuals, ranging from 0.28 to 32.38 km^2^ (Table 1). Consequently, the total distance flown in a night also varied greatly between individuals, with one individual regularly completing a 40-km round trip, while another individual typically flew 10 km from the roost to her foraging site and back, twice within a night (Fig 1). However, most foraging activity was focussed in substantially smaller core areas (CA, mean 2.9 ± 0.5 km^2^) which ranged from 0.97–5.82 km^2^ per individual (Table 1). There was no difference in home range or core area between bats who were highly selective in habitat choice (and therefore may commute further to access favoured sites) and those that used a greater variety of different habitat types (HR: F_1,9_ = 0.02, p = 0.87; CA: F_1,9_ = 0.48, p = 0.53). However, bats which roosted in buildings further from the plantation had significantly larger ranges than those roosting in buildings at the edge or within the plantation (Fig 2; HR: F_1,9_ = 48.18, p < 0.001; CA: F_1,9_ = 7.93, p = 0.02). Bats that roosted outside the plantation had home ranges that were up to five times larger than bats roosting inside the plantation (mean home range inside plantation: 5.2 ± 0.9 km^2^; mean home range outside plantation: 27.8 ± 5.2 km^2^).

Reproductive status had no influence on the duration and number of foraging bouts (Table B in S1 Appendix; Duration: F_3,7_ = 3.61, p = 0.27; Number of sessions: F_3,7_ = 1.04, p = 0.43). Whilst the duration of foraging sessions was marginally, but non-significantly, longer in 2014, there was no difference in the number of sessions between years (Table B in S1 Appendix; Duration: F_1,9_ = 4.31, p = 0.06; Number of sessions: F_1,9_ = 2.57, p = 0.13). The duration of foraging bouts was significantly longer when minimum temperatures were higher, although the number of foraging bouts was unchanged (Table B in S1 Appendix; Duration: 14.6 ± 5.1, R^2^ = 0.13, p = 0.006; Number of bouts: 0.02 ± 0.04, R^2^ = 0.01, p = 0.65).

### Compositional analysis

**Fig 3:**
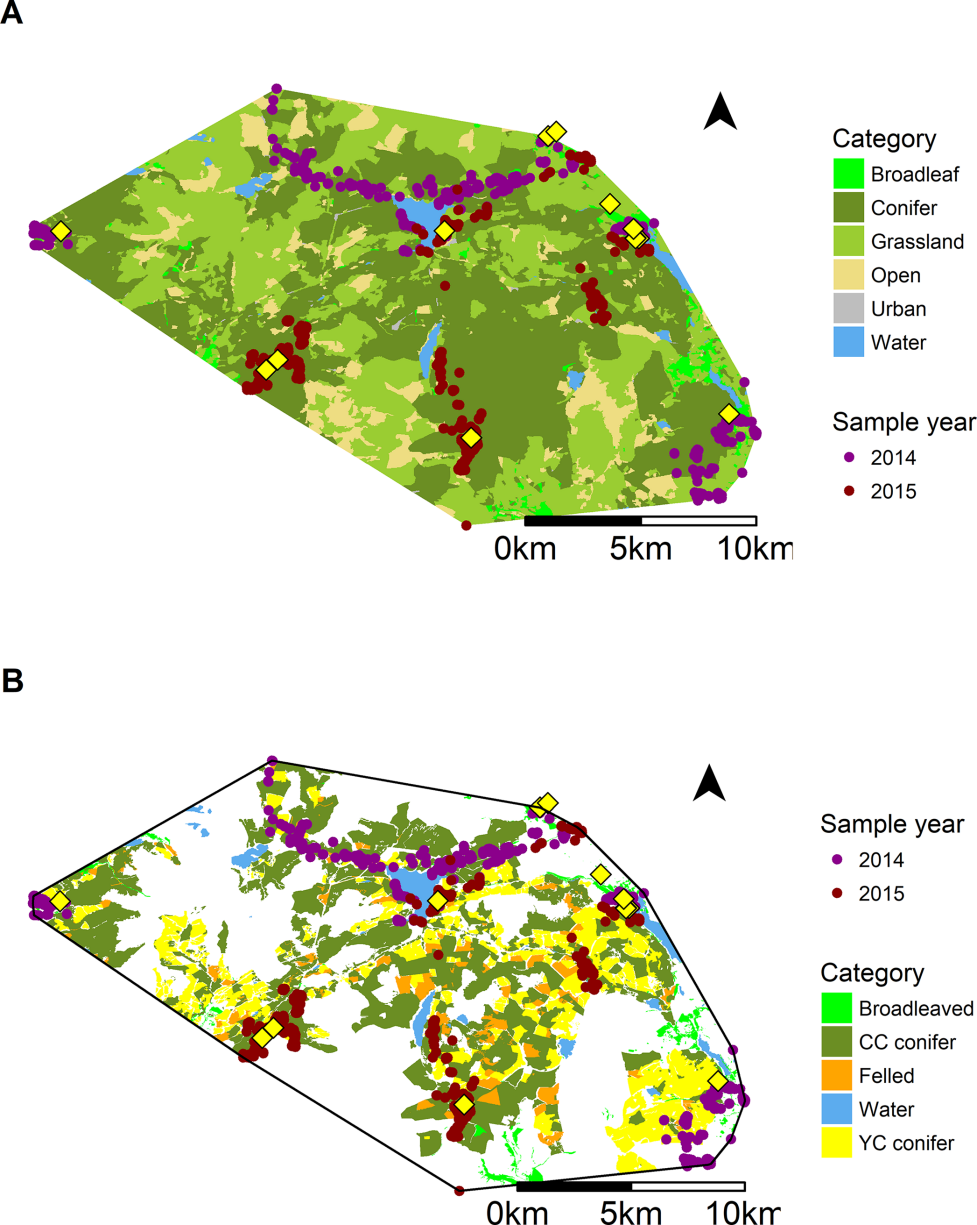
Total available habitat for *P*. *pygmaeus* (area inside minimum convex polygon formed around all fixes for all bats) and available habitat for (A) Broad resolution habitat map and (B) fine resolution forest management map. The white spaces on the fine resolution map indicate areas are upland, open areas which were not included in the fine scale analysis as the broad resolution analysis revealed very low use of these areas. Maternity roost locations for each bat indicated by a diamond. Tracking relocations are indicated by points.

Habitat use by bats was non-random and although individual bats were consistent in their use of core areas generally foraging along the same flight lines or within the same patches each night (Fig 3), there was little consistency between bats (df = 40, χ ^2^ = 855, p<0.001), therefore averaging across individuals was not appropriate. Instead we present and discuss individual Manly selection ratios (SR; Manly et al., 2007) and results of the eigen analysis (see Fig A and B in S1 Appendix for examples of both the *broad resolution* and *fine resolution* maps).

**Fig 4:**
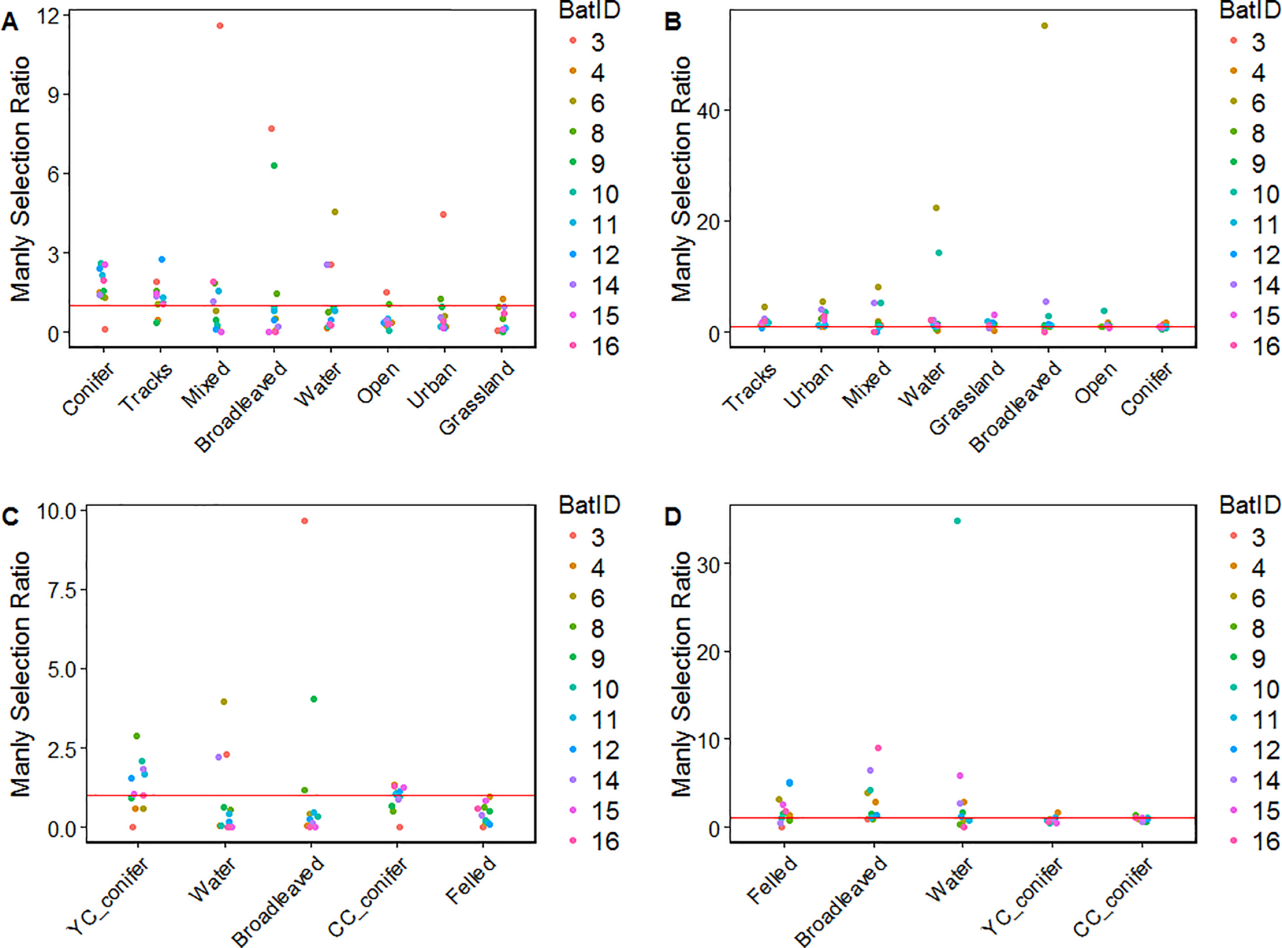
Individual Manly selection ratios for all bats at both the landscape scale using the broad (A) and fine (C) resolution habitat classifications, and local scale using the broad (B) and fine (D) resolution habitat classifications. Habitats are ranked from left to right according to the number of individuals which positively selected that habitat type. Points are coloured by bat ID. The red line indicates the point at which no selection or avoidance is occurring, above the red line indicates selection of that habitat type, below the red line indicates avoidance of that habitat type. YC conifer refers to young conifer, CC conifer refers to closed canopy conifer. See Table A in S1 Appendix for description of different stand types.

Nine of the 11 tagged bats showed some preference for conifers in their home range using the broad resolution map at the landscape scale, using it in a greater proportion than its availability despite being the dominant land cover type in the area. Somewhat surprisingly only four bats showed a preference for water (Table 2A, Fig 4A). Bat 3 was unusual in this study; at the landscape scale, she showed a strong preference for both broadleaf and mixed woodland habitat (Broadleaf: SR = 7.7; Mixed: SR = 11.6) and demonstrated clear avoidance of conifer and grassland habitats (Conifer: SR = 0.09; Grassland: SR = 0.03), meaning that she rarely entered within the plantation boundary.

**Table 2:** Broad resolution: Individual bat associations with each habitat type at (A) the landscape scale comparing habitat availability within each HR compared to overall availability and (B) the local scale comparing habitat availability in CA compared to HR for the Broad resolution land cover habitat descriptions. Selection ratios are represented as follows:—SR <0.25; — 0.25< SR < 0.50; - 0.50 < SR <0.75; ns 0.75 < SR < 1.25; + 1.25 < SR < 1.50; ++ 1.50 < SR < 2.5; +++ SR> 2.5.

(A) Bat associations at the landscape (home range) scale								
Bat ID	Human infrastructure	Broadleaf	Conifer	Open	Tracks	Mixed	Water	Grassland
3	+++	+++	—-	++	++	+++	+++	—-
4	—-	—-	+	—	—	—-	—-	ns
6	-	-	+	—	ns	ns	+++	ns
8	+	+	+	ns	++	++	ns	—
9	ns	+++	++	-	—	—	ns	—-
10	—-	ns	+++	—-	ns	—-	—-	—-
11	—-	ns	++	-	+	++	ns	—-
12	—-	—	++	—	+++	—-	—	—-
14	-	—-	+	—	+	ns	+++	ns
15	—-	—-	+++	—	+	—-	—	—-
16	—	—-	++	—	ns	++	—-	-
(B) Bat associations at the local (core area) scale								
BatID	Human infrastructure	Broadleaf	Conifer	Open	Tracks	Mixed	Water	Grassland
3	ns	ns	++	ns	ns	ns	ns	ns
6	+++	+++	-	ns	+++	+++	+++	ns
4	ns	—	++	++	++	++	—-	—
8	++	ns	ns	ns	ns	++	—	+
9	+	ns	-	ns	++	+	+	NA
10	+++	+++	-	+++	++	+++	+++	++
11	ns	ns	ns	ns	++	ns	ns	++
12	+	+	ns	ns	-	—-	+	++
14	+++	+++	-	ns	++	+++	++	-
15	++	NA	ns	-	++	NA	ns	+++
16	+++	—-	ns	ns	++	—-	++	ns

We used the *fine resolution* forest management map at the landscape scale (second order) to investigate bat associations with different management prescriptions within the plantation. Habitat preferences were ranked as following: Young conifer>>Water>>Broadleaf>>Closed canopy conifer>>Felled. Overall, habitat selection in the tracked bats is fairly weak (Table 3A). Again, two individuals strongly favour broadleaf areas while three favour water, two prefer closed canopy conifer and six show some selection for young conifer when comparing home ranges to available habitat (Table 3A, Fig 4C).

**Table 3:** Fine resolution: Individual bat associations with each habitat type at (A) the landscape scale comparing habitat availability within each HR compared to overall availability and (B) the local scale comparing habitat availability in CA compared to HR for the fine resolutionforest management habitat descriptions. Selection ratios are represented as follows:—SR <0.25; — 0.25< SR < 0.50; - 0.50 < SR <0.75; ns 0.75 < SR < 1.25; + 1.25 < SR < 1.50; ++ 1.50 < SR < 2.5; +++ SR> 2.5.

(A)Bat associations at the landscape (home range) scale					
BatID	Felled	Broadleaf	Closed canopy conifer	Young conifer	Water
3	—-	+++	—-	—-	++
4	ns	—-	+	-	—-
6	—-	—	ns	-	+++
8	-	ns	-	+++	-
9	-	+++	-	ns	-
10	—-	—	ns	++	—-
11	—-	—	ns	++	—
12	—-	—-	ns	++	—-
14	—	—-	ns	++	++
15	ns	—-	ns	ns	—-
16	-	—-	+	ns	—-
(B) Bat associations at the local (core area) scale					
BatID	Felled	Broadleaf	Closed canopy conifer	Young conifer	Water
3	—-	ns	NA	NA	—-
4	+	+++	ns	++	+++
6	+++	+++	ns	ns	-
8	ns	ns	+	ns	—
9	+	++	-	—	++
10	ns	+++	ns	—	+++
11	+++	ns	ns	ns	ns
12	+++	+	ns	ns	ns
14	—	+++	-	ns	+++
15	+++	NA	ns	—	+++
16	++	+++	ns	-	—-

At the local scale, comparing habitat use as determined by kernel density estimate, habitats were ranked as following for the *broad resolution* habitat classification: Tracks>>Urban>>Mixed>>Water>>Grassland>>Broadleaf>>Open>>Conifer (Table 2A). Three out eleven bats show a strong selection for broadleaf woodland within core areas compared to its overall availability within their home ranges, while three show little selection between habitat types and a further three selected mixed woodlands. Two bats preferentially selected water in their core areas, while bat 15 selected grassland over other habitat types (SR = 2.3) and bat 16 preferred urban and water habitats while avoiding broadleaf and mixed woodland habitats (Table 2B, Fig 4C).

Using the *fine resolution* classification at the local scale, half of the bats we tracked preferentially foraged over water compared to its availability in the core area, with four using water over all other habitat types. Bats 6, 11 and 12 preferentially foraged over felled stands compared to all other habitat types, while bat 9 foraged mostly over young conifer and bat 16 showed little association with any habitat feature. The habitat rankings had changed compared to selection in the home range: Water>>Felled>>Broadleaf>>Closed canopy conifer>>Young conifer (Table 3B, Fig 4D).

### Foraging area overlap

Most overlap in ranges of tracked bats occurred in their use of similar commuting routes to move around the plantation. Overlap between individual core areas was low, only occurring for 6 out of 55 potential pair combinations; the mean core area overlap was 11.7%. A high degree of overlap was seen between two pairs of bats (11 and 12; 15 and 16); both pairs shared a roost and were highly selective in their habitat choice. Frequent antagonistic interactions such as chasing behaviour were observed by fieldworkers while tracking.

## Discussion

These results demonstrate that *P*. *pygmaeus* makes widespread use of a commercial Sitka Spruce plantation for foraging during an energetically demanding period. The results from the radiotracking support previous work in the same plantation system using a combination of acoustic monitoring and trapping. Using acoustic monitoring, we found higher activity at recently felled stands compared to other stand management types [17], with activity increasing after felling [22]. Radiotracking confirmed preferential foraging over felled patches for a number of the individuals we tracked. There is a large, breeding population of P. pygmaeus in the study area as we identified several large roosts. While radiotracking is an intensive, costly and potentially invasive process, it provides unparalleled resolution of information about individual behaviour. Combining this with our findings in previous studies, we are able to confirm that *P*. *pygmaeus* use of commercial coniferous plantations does indeed seem to coincide with the presence of ephemeral but highly abundant invertebrate prey within an intensively managed, human dominated landscape. Whilst we are unable to estimate what proportion of bats in the 17 colonies located use plantations for foraging, the high density of individuals we recorded in building roosts within plantations suggests that *P*. *pygmaeus* use of the plantation matrix may be considerable. Our results differ from other studies carried out in predominantly agricultural environments with a low proportion of coniferous cover, which generally demonstrate avoidance of coniferous plantations [36,54,55]. In contrast, plantation cover dominated land use type in our study area. Although land cover within the plantations was heterogeneous when considering stand density and stand age, we found little evidence that bats preferentially selected broadleaf woodlands and avoided conifer. Habitat preferences were highly variable between individuals but consistent within individuals.

### Patterns of roost use in plantation dominated landscapes

All individuals roosted primarily in houses, and although we were unable to carry out full roost counts due to time constraints, at least two partial counts were carried out at all buildings we found bats roosting. In all but one case the roosts held more than 500 bats, with some roosts appearing to house far greater numbers of bats. Barlow and Jones [56] hypothesized that the large number of bats in *P*. *pygmaeus* roosts is due to the low number of suitable roosts near preferred foraging sites, and lack of appropriate roost space is a common feature of plantations managed for timber extraction [28]. Housing density is very low in our study area, limiting potential roost availability; the high proportion of buildings containing large roosts in Galloway implies a substantial population of bats resident in the area during the summer. Despite individuals being tagged in fairly close proximity to each other (often at the same site or within 5km of each other, they rarely roosted in the same building. It is possible that the density of bats in the Galloway plantation area is much higher than the population density in the agriculture dominated landscape in Aberdeenshire (Northern Scotland) surveyed by Nicholls and Racey [37,55], which may explain the much larger home ranges and contrasting habitat associations we found. No bats made use of coniferous trees of any species for roosting, although several individuals used deciduous tree roosts as night roosts and occasional day roosts. Tree roosts contained low numbers of bats, and bats were observed moving from maternity roosts to tree roosts after dawn, possibly suggesting that space in the maternity roost was limiting. Boughey et al. (2011) [6] found that *P*. *pygmaeus* roosts were more likely to be found in buildings nearer water and broadleaf woodland; in our study area most areas of broadleaf cover were in close proximity to human infrastructure (53% of broadleaf tree cover in the landscape was within 200m of a building, and 89% of broadleaf tree cover was within 1km of a building), and all the houses we found roosts in were within 100m of a watercourse. *Pipistrellus pygmaeus* also made use of abandoned buildings within the plantations including a small, derelict hut (since demolished), which supports our suggestion that roost space is lacking, and demonstrates the importance of ensuring surveys are undertaken if buildings need to be removed. Due to their ability to form large roosts in suitable buildings, *P*. *pygmaeus* are less dependent on suitable tree roosts than other species and can reach locally high population densities if foraging opportunities are sufficient. This flexibility in roost space use will allow them to access and monopolise potentially favourable foraging sites that lack suitable tree roosts required by other species (e.g. *Myotis nattereri;* [16]).

### Home range and core area size, overlap in ranges and antagonistic behaviour

Species habitat relationships are dependent on scale, with selection occurring at multiple spatial scales [57]. For highly mobile species such as bats, effective habitat management requires understanding of habitat associations at both the landscape (second order) scale and local, foraging (third order) scale [58]. In this study, home ranges were substantially larger than those reported from other studies [38,54,55], and varied considerably in size between bats, with the largest belonging to those roosting furthest away from the plantation. We recorded a mean home range of 9.51 km^2^, although the mean core area was much smaller at 2.9 km^2^. In contrast, studies in predominantly agricultural landscapes have recorded mean home ranges of 0.6 km^2^ [37,59] to 1.46 ± 0.27 km^2^ [54], with core foraging areas of 0.04 ± 0.02 km^2^ [54] to 0.25 km^2^ [55]. Large foraging ranges could be a response to low food availability (necessitating greater distances to find sufficient food), inter or intraspecific competition from other bats, or low roost availability [46,60]. Bats in areas with an abundance of both potential roosts and foraging areas tend to have smaller home ranges and greater overlap of individual ranges [61]; indeed, individuals roosting inside the plantation had smaller home ranges than those roosting outside the plantation. In this study, it is likely that the large difference in home range sizes we recorded compared to the above studies exists as a result of the distribution and abundance of available roosts, with bats roosting further from the plantation expending more energy to access profitable foraging sites [46]. Because flight is energetically expensive, the benefit of accessing these foraging areas must offset the costs of flight [46]. Clearly, *P*. *pygmaeus* is less constrained by behavioural (e.g. light avoidance) or ecomorphological (e.g. constraints on flying) factors than other, rarer species (e.g. *Rhinolophus hipposideros*; [49]) and the energetic cost of accessing plantation areas from more distant roosts is offset by the quality of available foraging areas. Commuting over large distances can even be a foraging strategy within itself when linked to the continuous intake of “aerial plankton”, as has been described for *Vespertilio murinus* [62] and *Eptesicus nilssonii* [63]. Use of commercial plantation landscapes by *P*. *pygmaeus* may therefore serve as an example of the resource dispersal theory [64]; larger home ranges are not a result of intraspecific competition but rather the dispersion and patchiness of resource availability (roosts and foraging patches; [64]). Group size is therefore determined by the heterogeneity and richness of the resource in question, while territory size is determined by the dispersion of the resource within the landscape [51]. Large home ranges allow bats to encompass sufficient foraging patches within their home range, particularly for individuals roosting outside the plantation, while the high density of invertebrates present in Sitka Spruce plantations (predominantly *Culicoides impuctatus*; [40]) can support the high density of *P*. *pygmaeus* recorded in roosts in and around the study area.

### Habitat associations

Bats across both years consistently used the same, few commuting routes to access plantations, primarily rivers and forest tracks. Linear landscape features such as forest tracks are known to be important features for bats for navigation and foraging, because they provide low structural complexity with high invertebrate abundance [31,60,65,66]. In plantation forests, where stands can be structurally dense, tracks may provide important flyways that allow bats to access foraging areas [7], as well as providing a suitable foraging habitat for edge adapted bats such as *P*. *pygmaeus* [25,60]. Most tracks in this study went through the plantation, therefore the strong association of all but two bats with tracks also reflects use of conifers. Most individuals appeared to preferentially select conifer above its availability at the landscape scale. This may reflect the large home ranges necessary for some bats, particularly those which roosted further from their preferred foraging sites.

At the local scale, using the fine resolution forest management map, bats showed little association with either young or closed canopy conifer but a strong preference for felled stands. This accords with findings from acoustic data where the lowest levels of activity were in and around 10 and 20-year old conifer stands [17]; activity alongside these stand types is likely to be a result of bat movement along forest tracks which are used by bats to access preferred foraging areas scattered within the plantation [65].

Individual bats were highly consistent between nights, visiting the same core areas repeatedly, but there was little consistency in habitat selection between bats at either the local or the landscape scale. Previous studies have identified high levels of habitat specificity for *P*. *pygmaeus*, particularly riparian and broadleaf habitats [6,36,37,67]. At the local, fine resolution scale, half of the bats tracked associated with both broadleaf, and to some extent water, in a greater proportion than its availability in their core area. However, most broadleaf patches within the plantation landscape are adjacent to river margins or buildings which often contained a roost, so it is difficult to disentangle the relative preference for these habitats. More surprising was the high degree of selection of felled stands at the local scale, despite most bats appearing to avoid felled stands at the landscape scale. Six bats preferentially selected felled stands in core foraging areas compared to their availability within individual home ranges and only two bats showed any avoidance at the local scale. Avoidance here may be an artefact of plantation management; at the landscape scale bats are likely to be selecting based on roost locations, which are patchily distributed within the landscape, and felling operations are often avoided near dwellings. While felled stands are also scattered throughout the landscape, they are ephemeral, which may also contribute to the far larger home ranges we found in this study compared to others [38,54,55] as individual bats may have to travel further to find newly felled patches. Felled patches, with large populations of *C*. *impuctatus* and low structural complexity, may represent an easily accessible and abundant food supply which certain individuals exploit [22,40].The difference between associations with felled patches at different spatial scales suggests that bats are responding to fine scale patchiness within superficially homogenous units (i.e. conifer plantations), and demonstrates the importance of investigating fine scale variations at the microhabitat level [68].

### Implications for conservation

Far from avoiding coniferous plantations, in this study, lactating female *P*. *pygmaeus* appear to actively select different management stages of Sitka spruce plantations for foraging, at an energetically demanding time of year. Roost availability in plantations, however, is very likely to be limiting. The high density of bats within maternity roosts suggests that there is a substantial *P*. *pygmaeus* population present in the area. Although we found no evidence of bats roosting in Sitka spruce or other coniferous trees, bats did use old or dead deciduous trees. Felling operations should aim to preserve these trees, with sufficient adjoining forest to render them less prone to windthrow. Retaining and expanding broadleaf patches, particularly in riparian areas will benefit bats by providing potential roosting areas important for harem formation in late summer [69]. However, as natural roosts are sparse in plantation landscapes, installing bat boxes along riparian zones, in remnant broadleaf patches and commuting routes into plantation areas may reduce pressure on maternity colonies and provide alternative roosts. There is evidence from both Australia and Europe that installing bat boxes in habitats with low roost availability often results in swift uptake [70,71]. As part of a parallel study [40], 50 bat boxes were erected in February 2016 in various locations around the study area, along known commuting routes and in areas near foraging patches. A survey was carried out to assess uptake of the boxes in the first year (September 2016); 131 bats (all *P*. *pygmaeus*, several mixed sex harems) were found in the boxes, over 90% of which had evidence of bat use [40]. The large number of harems found, even in boxes adjacent to each other is testament to the lack of suitable features for harems in plantation landscapes. However, the extent to which these boxes will be suitable for maternity roosts remains to be seen. It is highly unlikely that *P*. *pygmaeus* maternity colonies will form in boxes installed as part of this study (see Fig C and D in S1 Appendix for details of boxes used and placement), and this species is likely to continue to preferentially use building roosts [69]. Nevertheless, in Mediterranean wetlands *P*. *pygmaeus* formed maternity colonies in bat boxes; as roost availability is so low in plantation landscapes, further exploring the installation of different bat box types which may be appropriate for maternity roosts in plantation dominated landscapes is likely to benefit *P*. *pygmaeus* [71].

The strong preference for foraging over felled stands identified in this study has implications for the increasing installation of wind turbines in plantations. Establishing wind farms in plantation areas currently requires survey guidance for some protected species (e.g. hen harrier, *Circus cyaneus*), but there is no stipulation to survey for bats. The preferred management of phased felling and restocking up to keyholes (typically permanent open areas 50m from trees to turbine tip are retained while restocking occurs over the rest of the stand; [72]) is likely to result in a patchwork of felled areas linked by tracks which would be attractive foraging habitat for bats. Indeed, we have shown previously that bat activity increases in the short term post felling, particularly in smaller stands [22]. Wind turbines can cause both direct and indirect mortality to bats through collision and barrier effects resulting in changes to habitat use [73], although the extent to which such effects can exert population level impacts is likely to vary greatly between regions. Due to the perception of plantations being poor for bats, bat surveys pre-turbine installation are not a requirement, and pre-felling surveys are likely to underestimate bat activity post-felling. Therefore, considering the high density of *P*. *pygmaeus* roosts we identified in the plantation landscape, and the fact that *P*. *pipistrellus* also appear to be present in high numbers [17], installing wind turbines in Sitka Spruce plantations could pose a considerable risk to bat populations. More research is necessary to understand how *P*. *pipistrellus* and *N*. *leisleri*, both of which appear to have maternity colonies in our study area and responded positively to felling, use commercial plantation landscapes and decisions about siting wind turbines in upland plantations should take into account both pre- and post-felling bat populations in order to minimise the potential risk. Our results suggest that surveying for bats post felling for wind turbines should be required, particularly in low altitude areas, or at sites close to riverine habitat.; In a previous study, whilst bat activity increased following clear-felling, there was some evidence that this activity subsequently declined over time [22]. Further research to determine bat responses to harvesting operations could provide greater guidance for protecting bats.

### Conclusions

In landscapes dominated by Sitka Spruce plantations, plantations may form an important foraging habitat for a high density of *P*. *pygmaeus* during an energetically costly period, particularly in areas with a high abundance of nematoceran Diptera. This study provides further evidence of *P*. *pygmaeus’* adaptability; it is capable of flying much further distances than previously reported to reach foraging areas and able to adapt its foraging style to exploit opportunities offered by alternative habitats. However, this flexibility in exploiting ephemeral felled patches in the plantation landscape may render it vulnerable to alternative energy generation such as wind turbine installation.

## Supporting information

S1 Appendix Contains Tables A-B and Figs A–D. Table A: Stand characteristics for each management stage and stand features associated with management*Diameter at Breast Height = estimate of tree maturity. Table B: Summarised details of bat foraging sessions. Maximum and minimum temperatures are given in degrees celcius, and are the average maximum and minimum temperatures recorded during all foraging sessions for that bat. Fig A: Results of eigenanalysis using the broad resolution land cover map performed on individual selection ratios for (A) Second order habitat associations comparing habitat composition in bat home ranges compared to its availability and (B) Third order habitat associations comparing habitat composition in individual core areas compared to their home ranges. Numbers indicate individual bats, the direction and magnitude of the arrows show the direction and strength of the bats assocation with different habitat types and the clustering of the bats in space shows similarity between habitat selection. For example in A bat 4 is strongly associating with broadleaf, and is very different from the majority of other bats. Fig B: Results of eigenanalysis using the fine resolution forest management map performed on individual selection ratios for (A) Second order habitat associations comparing habitat composition in bat home ranges compared to its availability and (B) Third order habitat associations comparing habitat composition in individual core areas compared to their home ranges. Numbers indicate individual bats, the direction and magnitude of the arrows show the direction and strength of the bats assocation with different habitat types and the clustering of the bats in space shows similarity between habitat selection. For example in A bat 3 is strongly associating with broadleaf, and is very different from the majority of other bats. Fig C. Schwegler 1FF box with harem of *P*. *pygmaeus* inside. Fig D. Location of one of the boxes installed as part of this study. Boxes were put on trees in stands not included in felling schedules. Subsequent monitoring in 2017 found *P*. *pygmaeus* and *N*. *leisleri*
(DOCX)

S2 Appendix .csv file containing the proportion of different land cover types for both home range and core areas of all tagged bats. Landcover derived from the fine resolution forest map.
(CSV)

S3 Appendix .csv file containing the proportion of different land cover types for both home range and core areas of all tagged bats. Landcover derived from the broad resolution, landcover map.
(CSV)

S4 Appendix Reprojected tracking coordinates for all tracked bats coordinate system = British National Grid (BNG). (CSV)

## References

[1] Russo D, Cistrone L, Garonna AP, Jones G. Reconsidering the importance of harvested forests for the conservation of tree-dwelling bats. Biodivers Conserv. 2010;19: 2501–2515.

[2] Brockerhoff EG, Jactel H, Parrotta J a., Quine CP, Sayer J. Plantation forests and biodiversity: Oxymoron or opportunity? Biodivers Conserv. 2008;17: 925–951. 10.1007/s10531-008-9380-x

[3] Stephens SS, Wagner MMR. Forest plantations and biodiversity: a fresh perspective. J For. 2007;105: 307–313. Available: http://www.ingentaconnect.com/content/saf/jof/2007/00000105/00000006/art00012

[4] Humphrey JW. Benefits to biodiversity from developing old-growth conditions in British upland spruce plantations: a review and recommendations. Forestry. 2005;78: 33–53.

[5] Russo D, Billington G, Bontadina F, Dekker J, Dietz M, Jones G, et al Identifying key research objectives to make European forests greener for bats. Front Ecol Evol. 2016;4 10.3389/fevo.2016.00115

[6] Boughey KL, Lake IR, Haysom KA, Dolman PM. Effects of landscape-scale broadleaved woodland configuration and extent on roost location for six bat species across the UK. Biol Conserv. 2011;144: 2300–2310. 10.1016/j.biocon.2011.06.008

[7] Law B, Park K, Lacki MJ. Insectivorous Bats and Silviculture: Balancing Timber Production and Bat Conservation Bats in the Anthropocene: Conservation of Bats in a Changing World. Springer International Publishing, AG Switzerland; 2015 pp. 105–150.

[8] Borkin KM, Parsons S. Home range and habitat selection by a threatened bat in exotic plantation forest. For Ecol Manage. 2011;262: 845–852.

[9] Blakey R V., Law BS, Kingsford RT, Stoklosa J, Tap P, Williamson K. Bat communities respond positively to large-scale thinning of forest regrowth. J Appl Ecol. 2016; 10.1111/1365-2664.12691

[10] Adams MD, Law BS, French KO. Vegetation structure influences the vertical stratification of open- and edge-space aerial-foraging bats in harvested forests. For Ecol Manage. 2009;258: 2090–2100. 10.1016/j.foreco.2009.08.002

[11] Borkin KM, Parsons S. The importance of exotic plantation forest for the New Zealand long-tailed bat (Chalinolobus tuberculatus). New Zeal J Zool. 2010; 37–41. 10.1080/03014221003602190

[12] Borkin KM, Parsons S. Plantation forests are used by the lesser short-tailed bat, Mystacina tuberculata rhyacobia. New Zeal J Zool. 2010; 37–41. 10.1080/03014221003602174

[13] Patriquin KJ, Barclay RMR. Foraging by bats in cleared, thinned and unharvested boreal forest. J Appl Ecol. 2003;40: 646–657.

[14] Cistrone L, Altea T, Matteucci G, Posillico M, de Cinti B, Russo D. The effect of thinning on bat activity in italian high forests: The LIFE+ “ManFor C.BD.” experience. Hystrix. 2015;26: 125–131. 10.4404/hystrix-26.2–11477

[15] Charbonnier Y, Gaüzère P, van Halder I, Nezan J, Barnagaud J-Y, Jactel H, et al Deciduous trees increase bat diversity at stand and landscape scales in mosaic pine plantations. Landsc Ecol. 2016;31: 291–300. 10.1007/s10980-015-0242-0

[16] Mortimer G. Foraging, roosting and survival of natterer’s bats, Myotis nattereri, in a commercial coniferous plantation. Unpublished PhD thesis, University of St Andrews. 2006.

[17] Kirkpatrick L, Maher SJ, Lopez Z, Lintott PR, Bailey SA, Dent D, et al Bat use of commercial coniferous plantations at multiple spatial scales: Management and conservation implications. Biol Conserv. Elsevier B.V.; 2017;206: 1–10. 10.1016/j.biocon.2016.11.018

[18] Bender MJ, Castleberry SB, Miller DA, Bently Wigley T. Site occupancy of foraging bats on landscapes of managed pine forest. For Ecol Manage. 2015;336: 1–10. 10.1016/j.foreco.2014.10.004

[19] Morris AD, Miller DA, Kalcounis‐Rueppell MC. Use of forest edges by bats in a managed pine forest landscape. J Wildl Manage. 2010;74: 26–34.

[20] Mathews F, Richardson S, Lintott P, Hosken D. Understanding the risk to European Protected Species (bats) at onshore wind turbine sites to inform risk management. DEFRA Report WC0753; 2016.

[21] Jones G, Cooper-Bohannon R. Determining the potential ecological impact of wind turbines on bat populations in Britain Phase 1 Report Final. 2009; 11,12.

[22] Kirkpatrick L, Oldfield IF, Park K. Responses of bats to clear fell harvesting in Sitka Spruce plantations, and implications for wind turbine installation. For Ecol Manage. Elsevier B.V.; 2017;395: 1–8. 10.1016/j.foreco.2017.03.033

[23] Arnett EB, Baerwald EF, Mathews F, Rodrigues L, Rodríguez-durán A, Rydell J, et al Impacts of Wind Energy Development on Bats: A Global Perspective.: 295–323. 10.1007/978-3-319-25220-9

[24] Altringham JD, Hammond L, McOwat T. Bats: biology and behaviour. Oxford University Press,Oxford; 1996.

[25] Verboom B, Spoelstra K. Effects of food abundance and wind on the use of tree lines by an insectivorous bat, Pipistrellus pipistrellus. Can J Zool. 1999;77: 1393–1401. 10.1139/cjz-77-9-1393

[26] Heer K, Helbig-Bonitz M, Fernandes RG, Mello M a R, Kalko EK V. Effects of land use on bat diversity in a complex plantation-forest landscape in Northeastern Brazil. J Mammal. 2015;96: 720–731. 10.1093/jmammal/gyv068

[27] Rodríguez-San Pedro A, Simonetti JA. Does understory clutter reduce bat activity in forestry pine plantations? Eur J Wildl Res. 2014;61: 177–179. 10.1007/s10344-014-0871-7

[28] Burgar JM, Craig MD, Stokes VL. The importance of mature forest as bat roosting habitat within a production landscape. For Ecol Manage. Elsevier B.V.; 2015;356: 112–123. 10.1016/j.foreco.2015.07.027

[29] Borkin KM, Parsons S. Effects of Clear-Fell Harvest on Bat Home Range. PLoS One. 2014;9 10.1371/journal.pone.0086163 24465938 PMC3899175

[30] Borkin KM, O’Donnell C, Parsons S. Bat colony size reduction coincides with clear-fell harvest operations and high rates of roost loss in plantation forest. Biodivers Conserv. 2011;20: 3537–3548.

[31] Grindal SD, Brigham RM. Short-term effects of small scale habitat disturbance on activity by insectivorous bats. J Wildl Manage. 1998;62: 996–1003.

[32] Jung K, Kaiser S, Böhm S, Nieschulze J, Kalko EK V. Moving in three dimensions: effects of structural complexity on occurrence and activity of insectivorous bats in managed forest stands. J Appl Ecol. 2012;49: 523–531. 10.1111/j.1365-2664.2012.02116.x

[33] Dodd LE, Lacki MJ, Britzke ER, Buehler DA, Keyser PD, Larkin JL, et al Forest structure affects trophic linkages: how silvicultural disturbance impacts bats and their insect prey. For Ecol Manage. 2012;267: 262–270.

[34] Walsh AL, Harris S, Walsh L. Foraging habitat preferences of vespertilionid bats in Britain. J Appl Ecol. 1996;33: 508–518.

[35] Barlow KE, Briggs PA, Haysom KA, Hutson AM, Lechiara NL, Racey PA, et al Citizen science reveals trends in bat populations: The National Bat Monitoring Programme in Great Britain. Biol Conserv. 2015;182: 14–26. 10.1016/j.biocon.2014.11.022

[36] Davidson-Watts I, Walls S, Jones G. Differential habitat selection by Pipistrellus pipistrellus and Pipistrellus pygmaeus identifies distinct conservation needs for cryptic species of echolocating bats. Biol Conserv. 2006;133: 118–127. 10.1016/j.biocon.2006.05.027

[37] Nicholls B, Racey P. Habitat selection as a mechanism of resource partitioning in two cryptic bat species Pipistrellus pipistrellus and Pipistrellus pygmaeus. Ecography (Cop). 2006;29: 697–708. 10.1111/j.2006.0906–7590.04575.x

[38] Sattler T, Bontadina F, Hirzel A., Arlettaz R. Ecological niche modelling of two cryptic bat species calls for a reassessment of their conservation status. J Appl Ecol. 2007;44: 1188–1199. 10.1111/j.1365-2664.2007.01328.x

[39] Barlow KE. The diets of two phonic types of the bat Pipistrellus pipistrellus in Britain. J Zool London. 1997;243: 597–609. 10.1111/j.1469-7998.1997.tb02804.x

[40] Kirkpatrick L. Bat exploitation of Sitka Spruce plantations: Impacts of management on bats and nocturnal invertebrates. University of Stirling 2017.

[41] Hill D a., Greenaway FRANK. Effectiveness of an acoustic lure for surveying bats in British woodlands. Mamm Rev. 2005;35: 116–122. 10.1111/j.1365-2907.2005.00058.x

[42] Lintott PR, Fuentes-Montemayor E, Goulson D, Park KJ. Testing the effectiveness of surveying techniques in determining bat community composition within woodland. Wildl Res. 2013;40: 675–684. 10.1071/WR13153

[43] Kunz TH, Thomas DW, Richards GC, Tidemann CR, Pierson ED, Racey PA. Observational techniques for bats. Meas Monit Biol Divers Stand Methods Mamm. 1996; 105–114.

[44] Aldridge HDJN, Brigham RM. Load Carrying and Maneuverability in an Insectivorous Bat: a Test of the 5% " Rule " of Radio-Telemetry. J Mammal. 1988;69: 379–382. 10.2307/1381393

[45] White G., Garrott R. Analysis of wildlife radio-tracking data. London: Academic Press; 1990.

[46] O’Donnell CFJ. Large Home Range Size in the Ground Foraging Bat, Mystacina tuberculata, in Cold Temperate Rainforest, New Zealand. Acta Chiropterologica. 2015; 10.3161/150811014X687323

[47] Morton D, Rowland C, Wood C, Meek L, Marston C, Smith G, et al. Final Report for LCM2007—the new UK Land Cover Map. Countryside Survey Technical Report No. 11/07, NERC/Centre for Ecology & Hydrology; 2011.

[48] Harris S, Cresswell W., Forde P., Trewhella W., Woollard T, Wray S. Home-range analysis using radio-tracking data–a review of problems and techniques particularly as applied to the study of mammals. J Mammal. 1990;20: 97–123.

[49] Reiter G, Pölzer E, Mixanig H, Bontadina F, Hüttmeir U. Impact of landscape fragmentation on a specialised woodland bat, Rhinolophus hipposideros. Mamm Biol. 2013;78: 283–289. 10.1016/j.mambio.2012.11.003

[50] Bonaccorso FJ, Todd CM, Miles AC, Gorresen PM. Foraging range movements of the endangered Hawaiian hoary bat, Lasiurus cinereus semotus (Chiroptera: Vespertilionidae). J Mammal. 2015;96: 64–71. 10.1093/jmammal/gyu003

[51] Johnson DDP, Kays R, Blackwell PG, Macdonald DW. Does the resource dispersion hypothesis explain group living? Trends Ecol Evol. 2002;17: 563–570.

[52] Manly B., McDonald D., McDonald T., Wallace P. Resource Selection by Animals: Statistical Design and Analysis for Field Studies. Kluwer D, editor. Springer Science and Business Media; 2007.

[53] Calenge C, Dufour AB. Eigenalaysis of Selection Ratios from Animal Radio-Tracking Data. Ecology. 2006;87: 2349–2355. 16995635 10.1890/0012-9658(2006)87[2349:eosrfa]2.0.co;2

[54] Davidson-Watts I, Jones G. Differences in foraging behaviour between Pipistrellus pipistrellus (Schreber, 1774) and Pipistrellus pygmaeus (Leach, 1825). J Zool. 2005;268: 55–62. 10.1111/j.1469-7998.2005.00016.x

[55] Nicholls B, Racey PA. Contrasting home-range size and spatial partitioning in cryptic and sympatric pipistrelle bats. Behav Ecol Sociobiol. 2006;61: 131–142. 10.1007/s00265-006-0244-7

[56] Barlow KE. Roosts, echolocation calls and wing morphology of two phonic types of Pipistrellus pipistrellus. Int J Mamm bBology. 1999;64: 257–268.

[57] Chambers CL, Cushman S a., Medina-Fitoria A, Martínez-Fonseca J, Chávez-Velásquez M. Influences of scale on bat habitat relationships in a forested landscape in Nicaragua. Landsc Ecol. 2016; 1–20. 10.1007/s10980-016-0343-4

[58] Johnson DH, Prairie N. The Comparison of Usage and Availability Measurements for Evaluating Resource Preference. Ecology. 1980;61: 65–71. 10.2307/1937156

[59] Bartonicka T, Bielik A, Rehák Z, Bartonička T, Bielik A, Řehák Z. Roost Switching and Activity Patterns in the Soprano Pipistrelle, Pipistrellus pygmaeus, during Lactation. Ann Zool Fennici. Finnish Zoological and Botanical Publishing Board; 2008;45: 503–512. 10.5735/086.045.0605

[60] Ciechanowski M. Habitat preferences of bats in anthropogenically altered, mosaic landscapes of northern Poland. Eur J Wildl Res. 2015;61: 415–428. 10.1007/s10344-015-0911-y

[61] August TA, Nunn MA, Fensome AG, Linton DM, Mathews F. Sympatric Woodland Myotis Bats Form Tight-Knit Social Groups with Exclusive Roost Home Ranges. PLoS One. 2014;9 10.1371/journal.pone.0112225 25356770 PMC4214762

[62] Rydell J. Nordic Society Oikos The Diet of the Parti-Coloured Bat Vespertilio murinus in Sweden Author (s): Jens Rydell Published by: Wiley on behalf of Nordic Society Oikos Stable URL: http://www.jstor.org/stable/3683190 The diet of the parti-coloured bat Vesp. 2017;15: 195–198.

[63] Haupt M, Menzler S, Schmidt S. Flexibility of Habitat Use in Eptesicus nilssonii: Does the Species Profit from Anthropogenically Altered Habitats? Am Soc Mammal. 2006;87: 351–361.

[64] Carr GM, Macdonald DW. The sociality of solitary foragers: a model based on resource dispersion. Anim Behav. 1986;34: 1540–1549.

[65] Hein CD. Bat activity and roost-site selection on an intensively managed pine landscape with forested corridors in the Lower Coastal Plain of South Carolina. Unpublished PhD thesis, Texas A&M University. 2008.

[66] Schnitzler HU, Kalko EK V, Miller L, Surlykke A. How the bat, Pipistrellus kuhli, hunts for insects In: Nachtigall PE, Moore PWB, editors. Animal Sonar Processes and Performance. New York: Plenum Press; 1988 pp. 619–624.

[67] Russ J., Montgomery W. Habitat associations of bats in Northern Ireland: implications for conservation. Biol Conserv. 2002;108: 49–58. 10.1016/S0006-3207(02)00089-7

[68] Arrizabalaga-Escudero A, Napal M, Aihartza J, Garin I, Alberdi A, Salsamendi E. Can pinewoods provide habitat for a deciduous forest specialist? A two-scale approach to the habitat selection of Bechstein’s bat. Mamm Biol. 2013;

[69] Park KJ, Masters E, Altringham JD. Social structure of three sympatric bat species (Vespertilionidae). J Zool. 1998;244: 379–389. 10.1017/S0952836998003094

[70] López-Baucells A, Puig-Montserrat X, Torre I, Freixas L, Mas M, Arrizabalaga A, et al Bat boxes in urban non-native forests: a popular practice that should be reconsidered. Urban Ecosyst. Urban Ecosystems; 2016; 10.1007/s11252-015-0519-8

[71] Flaquer C, Torre I, Ruiz-Jarillo R. The value of bat-boxes in the conservation of Pipistrellus pygmaeus in wetland rice paddies. Biol Conserv. 2006;128: 223–230. 10.1016/j.biocon.2005.09.030

[72] Anon. Wind farm proposals on afforested sites–advice on measures to minimise attractiveness to hen harrier, merlin and short-eared owl. Scottish Natural Heritage; 2015.

[73] Voigt CC, Kingston T. Bats in the Anthropocene: Conservation of Bats in a Changing World. Bats in the Anthropocene: Conservation of Bats in a Changing World. 2015 10.1007/978-3-319-25220-9

