# Supplementary material for: Flexible foraging strategies in Pipistrellus pygmaeus in response to abundant but ephemeral prey
Source: PLoS One. 2018 Oct 4;13(10):e0204511. doi: 10.1371/journal.pone.0204511 (PMC6171852; doi:10.1371/journal.pone.0204511)
Supplement: S1 Appendix — Contains Tables A-B and Figs A–D. Table A: Stand characteristics for each management stage and stand features associated with management*Diameter at Breast Height = estimate of tree maturity. Table B: Summarised details of bat foraging sessions. Maximum and minimum temperatures are given in degrees celcius, and are the average maximum and minimum temperatures recorded during all foraging sessions for that bat. Fig A: Results of eigenanalysis using the broad resolution land cover map performed on individual selection ratios for (A) Second order habitat associations comparing habitat composition in bat home ranges compared to its availability and (B) Third order habitat associations comparing habitat composition in individual core areas compared to their home ranges. Numbers indicate individual bats, the direction and magnitude of the arrows show the direction and strength of the bats assocation with different habitat types and the clustering of the bats in space shows similarity between habitat selection. For example in A bat 4 is strongly associating with broadleaf, and is very different from the majority of other bats. Fig B: Results of eigenanalysis using the fine resolution forest management map performed on individual selection ratios for (A) Second order habitat associations comparing habitat composition in bat home ranges compared to its availability and (B) Third order habitat associations comparing habitat composition in individual core areas compared to their home ranges. Numbers indicate individual bats, the direction and magnitude of the arrows show the direction and strength of the bats assocation with different habitat types and the clustering of the bats in space shows similarity between habitat selection. For example in A bat 3 is strongly associating with broadleaf, and is very different from the majority of other bats. Fig C. Schwegler 1FF box with harem of P. pygmaeus inside. Fig D. Location of one of the boxes installed as part of this study. Boxe [file pone.0204511.s001.docx]

S1 Table A: Description of stand types

Stand characteristics for each management stage and stand features associated with management. *Diameter at Breast Height – estimate of tree maturity

| Stand Age | Management Stage | Key stand features |
| --- | --- | --- |
| 40 – 60 years | Mature | Occasionally thinned, stand density between 500 and 2200 stems ha^-1^, average stand density: 1267 stems ha^-1^, canopy closure between 80 and 100%, average closure 99% |
| 20 – 40 years | Thin | Trees more densely packed, losing midstem branches and some trees dying off (self thinned). Occasionally thinned through management. Stand density between 600 – 2800 stems ha^-1^, average stand density: 1624 stems ha^-1^. Canopy closure between 50 and 100%, average closure: 95% |
| 10 – 20 years | Thicket | Very dense, retain midstem branches, no undergrowth. Stand density between 300 – 3000 stems ha^-1^, average stand density: 1850 stems ha^-1^. Canopy closure between 16 and 100%, average closure: 69% |
| 5 – 10 years | Young | Small, nearly all trees < 7cm DBH*, no canopy closure, lots of vegetation and ground cover |
| Clearfell | Felled < 5 years ago | Lots of dead wood and brash, standing water and undergrowth |

S1 Table B: Summarised details of bat foraging sessions. Maximum and minimum temperatures are given in degrees celcius, and are the average maximum and minimum temperatures recorded during all foraging sessions for that bat.

|  |  |  |  |  |  |  |  |
| --- | --- | --- | --- | --- | --- | --- | --- |
| Animal ID | Study year | Reproductive status | Number of nights | Total No Foraging sessions | Average foraging session length (mins) | Average maximum temperature | Average minimum temperature |
| 3 | 2014 | Lac | 5 | 11 | 177 ± 13.8 | 12 | 10 |
| 4 | 2014 | Lac | 3 | 4 | 290 ± 60.9 | 15 | 14 |
| 6 | 2014 | Lac | 5 | 6 | 336 ± 38.7 | 15 | 12 |
| 8 | 2014 | Plac | 5 | 6 | 277 ± 63.4 | 14 | 12 |
| 9 | 2014 | NA (Juvenile) | 3 | 4 | 113 ± 15.8 | 12 | 9 |
| 10 | 2015 | Preg | 4 | 6 | 165 ± 21.1 | 12 | 11 |
| 11 | 2015 | Preg | 4 | 8 | 134 ± 22.9 | 14 | 12 |
| 12 | 2015 | Preg | 4 | 9 | 122 ± 38.5 | 12 | 9 |
| 14 | 2015 | Lac | 4 | 7 | 134 ± 8.3 | 12 | 8 |
| 15 | 2015 | Lac | 6 | 10 | 207 ± 12.5 | 12 | 9 |
| 16 | 2015 | PLac | 5 | 8 | 176 ± 68.1 | 11 | 8 |

Eigen analysis results.


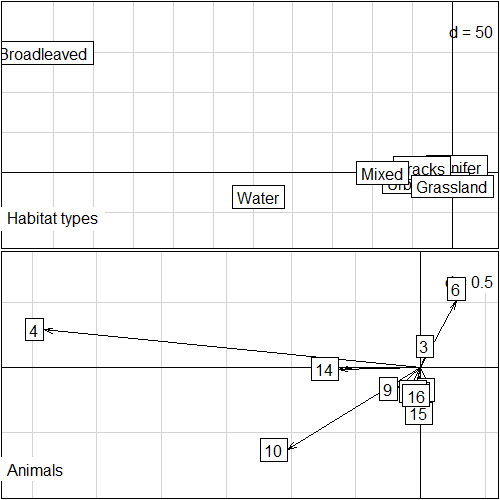

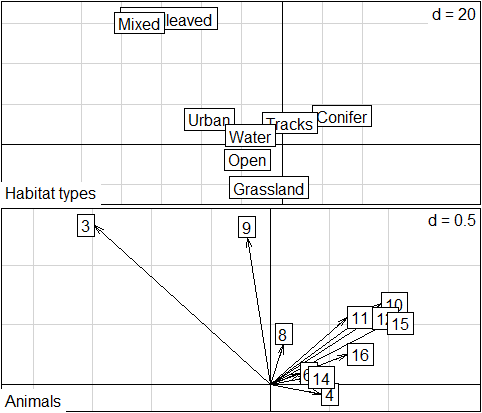


S1 Fig A: Results of eigenanalysis using the broad resolution land cover map performed on individual selection ratios for (A) Second order habitat associations comparing habitat composition in bat home ranges compared to its availability and (B) Third order habitat associations comparing habitat composition in individual core areas compared to their home ranges. Numbers indicate individual bats, the direction and magnitude of the arrows show the direction and strength of the bats assocation with different habitat types and the clustering of the bats in space shows similarity between habitat selection. For example in A bat 4 is strongly associating with broadleaf, and is very different from the majority of other bats.


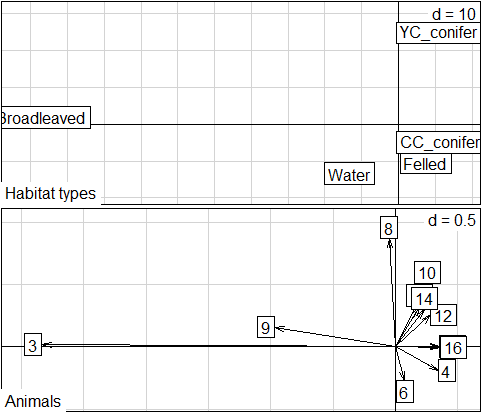

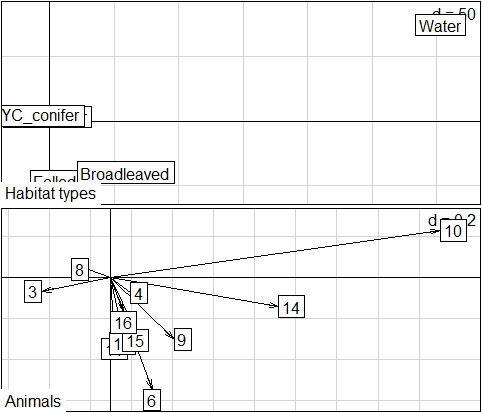


S1 Fig B: Results of eigenanalysis using the fine resolution forest management map performed on individual selection ratios for (A) Second order habitat associations comparing habitat composition in bat home ranges compared to its availability and (B) Third order habitat associations comparing habitat composition in individual core areas compared to their home ranges. Numbers indicate individual bats, the direction and magnitude of the arrows show the direction and strength of the bats assocation with different habitat types and the clustering of the bats in space shows similarity between habitat selection. For example in A bat 3 is strongly associating with broadleaf, and is very different from the majority of other bats.

**S3**: Description of bat boxes installed in Galloway forest park (August 2015 and February 2016). Pictures reproduced with permission from John Martin.

All boxes installed as part of this work were Schwegler 1FF’s and a further 9 2F Schwegler boxes were installed as part of mitigation for removal of an old hide. Bat use of the old hide was identified due to tracking work carried out as part of this project. Boxes were installed in august 2015 (2F) and February 2016 (1FF). Boxes were checked in September 2016.All box checks were carried out under license


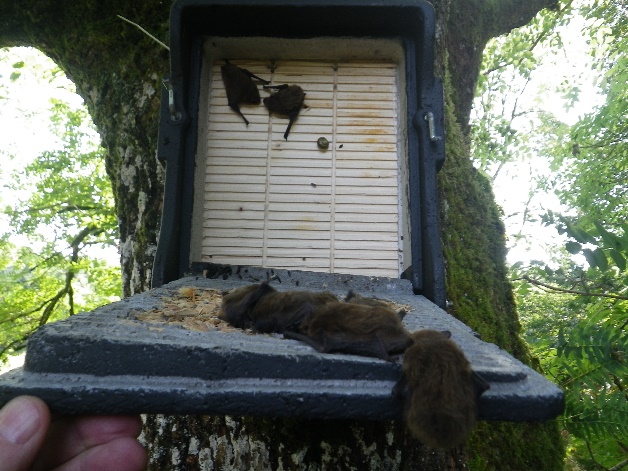

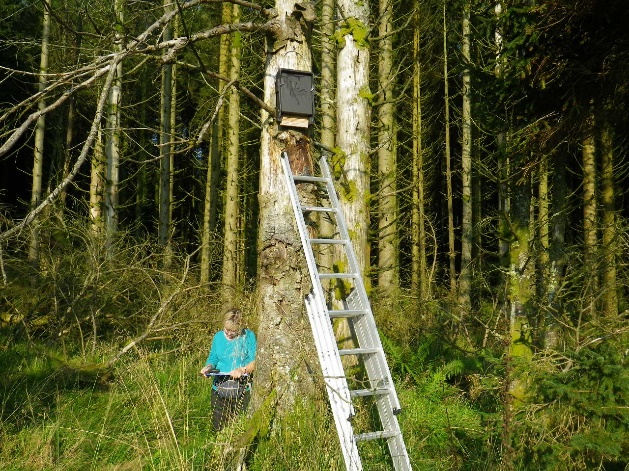


S1 Fig C. Schwegler 1FF box with harem of *P. pygmaeus* inside. B. Location of one of the boxes installed as part of this study. Boxes were put on trees in stands not included in felling schedules. Subsequent monitoring in 2017 found *P. pygmaeus* and *N. leisleri*
